# Influence of Skeletal Muscle Carnosine Content on Fatigue during Repeated Resistance Exercise in Recreationally Active Women

**DOI:** 10.3390/nu9090988

**Published:** 2017-09-07

**Authors:** Alyssa N. Varanoske, Jay R. Hoffman, David D. Church, Ran Wang, Kayla M. Baker, Sarah J. Dodd, Nicholas A. Coker, Leonardo P. Oliveira, Virgil L. Dawson, David H. Fukuda, Jeffrey R. Stout

**Affiliations:** 1 Institute of Exercise Physiology and Wellness, Educational and Human Sciences, Sport and Exercise Science, University of Central Florida, 12494 University Blvd., Orlando, FL 32816, USA; alyssa.varanoske@ucf.edu (A.N.V.); david.church@ucf.edu (D.D.C.); ran.wang@ucf.edu (R.W.); kayla.baker@ucf.edu (K.M.B.); sdodd1@knights.ucf.edu (S.J.D.); n.coker0418@knights.ucf.edu (N.A.C.); david.fukuda@ucf.edu (D.H.F.); jeffrey.stout@ucf.edu (J.R.S.); 2 Department of Internal Medicine, College of Medicine, University of Central Florida, Orlando, FL 32816, USA; leonardo.oliveira@ucf.edu (L.P.O.); virgil.dawson@ucf.edu (V.L.D.)

**Keywords:** intracellular buffering capacity, muscular acidosis, fatigue, dietary protein intake, histidine dipeptides

## Abstract

Carnosine is a naturally occurring intramuscular dipeptide that is thought to attenuate fatigue during high-intensity exercise. Carnosine content is influenced by various factors, including gender and diet. Despite research reporting that carnosine content is lower in women compared to men and lower in vegetarians compared to omnivores, no investigations have examined carnosine content in women based on dietary protein intake and its effect on muscle fatigue. Twenty recreationally active women were assigned to either a high (HI; *n* = 5), moderate (MOD; *n* = 10), or low (LO; *n* = 5) group based upon intramuscular carnosine content of the vastus lateralis. Each participant underwent two unilateral maximal voluntary isometric contractions (MVIC) of the knee extensors separated by an isokinetic exercise protocol consisting of five sets of 50 repeated maximal unilateral contractions. Magnitude-based inferences were used to analyze group differences. Percent decline in rate of force development and peak torque (PT) during the MVICs and changes in PT and mean torque during the muscle-fatiguing protocol were lower in HI compared to both MOD and LO. Additionally, absolute and relative dietary protein intake were greater in HI compared to MOD or LO. Results indicated that greater intramuscular carnosine content was reflective of greater dietary protein intake and that individuals with higher carnosine content displayed a greater attenuation of fatigue compared to those with lower carnosine.

## 1. Introduction

Carnosine (β-alanyl-l-histidine) is a naturally occurring dipeptide molecule that exists in high concentrations in skeletal muscle [1]. Carnosine is composed of two amino acids; l-histidine and β-alanine, with the latter being rate-limiting to carnosine formation in vivo [1,2]. Muscle carnosine has been postulated to be ergogenic due to the pKa of the imidazole ring of histidine, which is ideal for buffering H^+^ in physiological ranges [3]. Furthermore, when attached to β-alanine, the acid dissociation constant of histidine increases slightly [4], allowing it to act as a buffer during muscular contraction and fatigue, therefore improving sporting performance by delaying muscular contraction-induced acidosis. Recent investigations suggest that intramuscular carnosine may also act as a diffusible Ca^2+^/H^+^ exchanger at the sarcomere, augmenting skeletal muscle force production [5,6]. Because carnosine can bind both H^+^ and Ca^2+^, engaging in high-intensity activities results in an increase in H^+^ accumulation and in H^+^ binding to carnosine. This induces Ca^2+^ unloading at the sarcomere, and thus increases cross-bridge formation and force production [5,6]. In support of this, Suzuki et al. [7] demonstrated that males with higher carnosine content had greater power output during the latter phases of a 30-s Wingate test compared to those with lower carnosine. Additionally, Baguet and colleagues [8] showed that rowers with higher carnosine levels had faster split times in the second and third 500 m splits of a 2000 m race (approximately 1.5–4.5 min into the race). Taken together, increased muscle carnosine concentration appears to be most beneficial for increasing performance in anaerobic events due to the buffering of high levels of H^+^ ions produced during high-intensity activities.

Skeletal muscle carnosine content is dependent on various individual factors including age, gender, diet, muscle fiber type distribution, and training status [4]. Research investigating the effects of gender on carnosine content has shown that men have approximately 20–25% greater carnosine compared to women [9,10,11], which may be a result of the greater percentage of Type II muscle fibers in men compared to women [12]. Carnosine content in Type II fibers is approximately 30–100% greater than carnosine content in Type I fibers [10,13,14], further demonstrating the importance of carnosine during high-intensity activities. Research has also shown that intramuscular carnosine content is lower in vegetarians, which is most likely a result of β-alanine being present in higher quantities in meat and animal products [10]; however, previous research did not observe a significant correlation between the amount of dietary β-alanine consumption and resultant carnosine content in omnivorous men [10]. 

Most of the studies investigating carnosine content and its relationship to anaerobic performance have used β-alanine supplementation protocols to elicit increases in intramuscular carnosine content [13,15,16,17]. Generally, β-alanine supplementation has resulted in positive effects on anaerobic sporting performance, demonstrating that it is most beneficial for activities lasting 1–4 min, which may be due to the simultaneous increase in muscle carnosine content and therefore increased buffering capacity [4,8,13,16]. However, very few studies have investigated the effects of skeletal muscle carnosine content on measures of fatigue, and even fewer studies have been conducted in women. To the best of our knowledge, no investigators have examined the effects of muscle carnosine content on exercise performance in recreationally active females without β-alanine supplementation. Therefore, the purpose of this study was to examine the effects of resting muscle carnosine content on isokinetic and isometric exercise performance in recreationally active females. A secondary purpose of this investigation was to examine dietary protein intake and its relationship to intramuscular carnosine content in recreationally active women.

## 2. Materials and Methods

### 2.1. Experimental Design

**Figure 1:**
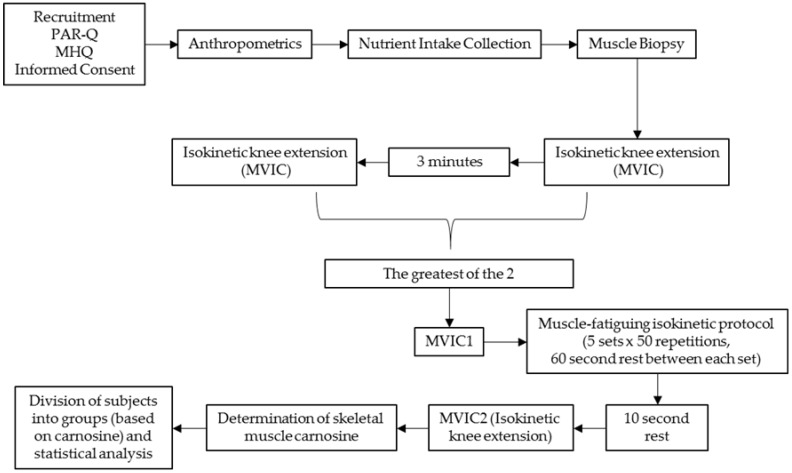
Study design. PAR-Q: Physical Activity Readiness Questionnaire; MHQ: Medical History Questionnaire; MVIC: Maximal Voluntary Isometric Contraction.

The study was conducted within the Human Performance Laboratory (HPL) of the Institute of Exercise Physiology and Wellness at the University of Central Florida. All study procedures occurred in a single visit. During the experimental testing day, participants arrived to the HPL and initially underwent anthropometric measures followed by a muscle biopsy performed on their left vastus lateralis (VL) muscle. Thirty minutes following the muscle biopsy, participants engaged in two separate isometric knee extensor testing sessions, followed by a muscle-fatiguing isokinetic knee extensor protocol, which was then followed by another isometric knee extensor protocol. All performance testing was conducted on the participant’s right leg. Prior to each testing session, participants were instructed to fast for a minimum of two hours and avoid lower body physical activity for 48 h prior to testing. The study design is presented in Figure 1.

### 2.2. Participants

Twenty-one recreationally active women were recruited for this investigation. Participants were instructed to maintain normal food and exercise habits throughout the period leading up to the experimental trial. This investigation was approved by the New England Institutional Review Board for human subjects (NE IRB# 16-086, approved 30 March 2016), and all procedures were in accordance with the ethical standards of the 1964 Helsinki Declaration and its later amendments. Following an explanation of all procedures, risks, and benefits, each participant provided their written informed consent to participate in the study. All participants were required to be free of any physical limitations (as determined by medical history questionnaire and Physical Activity Readiness Questionnaire (PAR-Q)), and were deemed to be physically active, as defined by American College of Sports Medicine standards. Participants were separated into high (HI), moderate (MOD), or low (LO) groups based on their skeletal muscle carnosine content. All performance assessments were conducted prior to allocating participants to a specific group.

### 2.3. Anthropometric Measurements

Anthropometric measurements were assessed for each participant during their visit to the laboratory. Upon arrival to the laboratory, subjects were instructed to void their bladder in order to properly assess body composition. Height (±0.1 cm) and body mass (±0.1 kg) were determined using a Health-O-Meter Professional scale (Model 500 KL, Pelstar, Alsip, IL, USA). Body composition was assessed via air displacement plethysmography (BodPod^®^, COSMED, Chicago, IL, USA).

### 2.4. Nutrient Intake and Dietary Analysis

Participants were instructed to maintain their normal dietary intake habits prior to the investigation. Total energy and macronutrient intakes were monitored using recorded food logs during the 72-h period prior to the visit. The FoodWorks Dietary Analysis Software, Version 13 (The Nutrition Company, Long Valley, NJ, USA) was used to analyze dietary recalls.

### 2.5. Muscle Biopsies

Prior to testing, all participants were instructed to wear shorts on testing day to expose the upper portion of their thigh. Biopsy methods were previously described by Church et al. [18]. Briefly, fine-needle muscle biopsies were obtained from the VL muscle of the left leg at 50% of the straight line distance between the lateral border of the patella and the greater trochanter of the femur [19]. A B-mode, linear probe ultrasound (General Electric LOGIQe, Wauwatosa, WI, USA), coated with transmission gel (Aquasonic^®^ 100, Parker Laboratories, Inc., Fairfield, NJ, USA) was used to determine muscle thickness and subcutaneous adipose tissue thickness at the aforementioned location on the leg to provide guidance prior to biopsy procedures. The biopsy area was washed with antiseptic soap and cleaned with rubbing alcohol. A small area of the clean skin approximately 2 cm in diameter was then anesthetized with a 2.0 mL subcutaneous injection of Lidocaine. The biopsy site was further cleansed by swabbing the area with betadine. Once anesthetized, a spring-loaded reusable microbiopsy instrument with a disposable 14-gauge needle (Argon Medical Devices Inc., Plano, TX, USA) was inserted into the skin at an approximate depth of 1–2 cm to extract the muscle sample. Approximately 5–6 muscle samples were extracted from each participant on each occasion, with the goal of obtaining about 15–20 mg of total wet tissue weight. After removal, each muscle sample was transferred to a petri dish placed on ice to trim adipose tissue from the muscle specimens. The remaining muscle specimens were then immediately frozen in liquid nitrogen and stored at −80 °C for later analysis.

### 2.6. Preparation of Skeletal Muscle Tissue for High-Performance Liquid Chromatography (HPLC) Analysis

Muscle biopsy samples were homogenized with 3 volumes of 0.01 N hydrochloric acid (Sigma-Aldrich, St. Louis, MO, USA), and subsequently centrifuged at 4 °C for 20 min at 10,000 rpm. Muscle homogenates and plasma were deproteinized with 3 volumes of acetonitrile (BDH VWR Analytical, Radnor, PA, USA), and left to stand at 4 °C for 20 min. Then the sample was centrifuged at 4 °C for 10 min at 10,000 rpm. The supernatant was collected and subsequently analyzed.

### 2.7. Determination of Skeletal Muscle Carnosine

The experimental methods were performed as described by Mora and colleagues [20]. Calibration standards were prepared in the range of 0.1–5 mM by dilution of a stock 10 mM solution. Chromatography was performed on an Agilent Infinity 1260 HPLC (Agilent Technologies, Santa Clara, CA, USA), and separation was carried out using an Atlantis hydrophilic interaction chromatography (HILIC) silica column (4.6 × 150 mm, 3 μm) from Waters (Milford, MA, USA) at room temperature. Mobile phase consisted of solvent A, containing 0.65 mM ammonium acetate (Sigma-Aldrich, St. Louis, MO, USA), pH 5.5, in water/acetonitrile (25:75), and solvent B, containing 4.55 mM ammonium acetate, pH 5.5, in water/acetonitrile (70:30). Solvents were filtered through a 0.22 μm membrane filter and degassed prior to the analytical run. The separation conditions were a linear gradient from 0 to 100% of solvent B in 13 min at a flow rate of 1.4 mL·min^−1^. The column was equilibrated for 10 min under initial conditions before each injection. The separation was monitored using a diode array detector at a wavelength of 214 nm for carnosine and histidine. Peak areas were correlated to compound concentration by interpolation in the corresponding calibration curve. Duplication of retention times for a known standard was used to verify column equilibrium prior to analysis. The average intra-assay coefficient of variation (CV) of carnosine was 1.37%, and the inter-assay CV was 5.45%.

### 2.8. Isometric Testing and Isokinetic Muscle-Fatiguing Protocol

Considering that participants were reporting to the laboratory on a two-hour minimum fast, an 8-ounce carbohydrate-containing beverage (60 calories, 16 g carbohydrates, 0 g fat, and 0 g protein) was provided to each participant following the muscle biopsy, 30 min prior to the isokinetic muscle-fatiguing protocol. The isometric testing and isokinetic muscle-fatiguing protocol have been previously described [18]. Briefly, unilateral maximal voluntary isometric contractions (MVIC) and an isokinetic muscle-fatiguing protocol were performed on an isokinetic dynamometer (System 4, Biodex Medical System, Inc., New York, NY, USA). To avoid any residual effects of the muscle biopsy, the right leg of each participant was tested on the isokinetic dynamometer. The lower portion of the leg was secured to the dynamometer arm just above the medial and lateral malleoli. Participants were seated in the dynamometer with a hip angle of 110° and strapped to the chair at the waist, shoulders, and across the left thigh. Chair and dynamometer settings were adjusted for each participant to properly align the axis of rotation of the knee with the lateral condyle of the femur. Range of motion was assessed for each participant. All participants were able to achieve a range of motion of 90–170° without discomfort. The gravity effect of moment was measured at 120° of knee flexion (180° representing full extension) and subsequently corrected during testing [21].

The isokinetic muscle-fatiguing protocol consisted of 5 × 50 maximal voluntary isokinetic unilateral knee extensions at a constant angular velocity of 180°∙s^−1^. Each contraction was initiated from a position of 90° knee flexion and was continued to the participant’s normal endpoint range of motion. After each extension, the lower leg was passively returned to the start position at 90°∙s^−1^ (~1.5 s for a full cycle). Each bout of 50 repetitions was separated by a 60-s recovery period. Subjects were encouraged during the first three repetitions to make sure that they were contracting maximally from the start of each bout. Mean torque (MT) and peak torque (PT) were recorded for each of the 250 repetitions. The average MT and PT for each set of 50 repetitions, as well as the change in average MT and PT from the first set to all other sets, were then further analyzed. Reliability statistics for the MT and PT during the muscle-fatiguing protocol were completed on a separate sample of participants, separated by a period of two weeks. The intraclass correlation (ICC) for MT using model (3,1) was ICC_3,1_ = 0.889, and the standard error of measurement (SEM) for MT using model (3,1) was SEM_3,1_ = 7.20 Nm. For PT, the following reliability statistics were collected: ICC_3,1_ = 0.923, SEM_3,1_ = 7.54 Nm.

Two MVICs were performed prior to the isokinetic muscle-fatiguing protocol, separated by a period of three minutes. Of the two MVICs performed prior to the isokinetic muscle-fatiguing protocol, the one that produced the greatest PT (MVIC1) was saved and used for later analysis. Additionally, one MVIC was performed 10 s after the final set of the muscle-fatiguing protocol was completed (MVIC2). During these tests, the knee angle was fixed at 110°. PT and peak rate of force development (RFD) were determined from each MVIC. Percent decline (% DEC) in PT and RFD from MVIC1 to MVIC2 was calculated to evaluate the effects of muscle fatigue using the following equation:

% DEC from MVIC1 to MVIC2 = ((MVIC1 − MVIC2))/MVIC1 × 100
(1)

Reliability statistics for MVIC PT and RFD were completed on a separate sample of participants, separated by a period of two weeks. For PT, the following reliability statistics were collected: ICC_3,1_ = 0.875, SEM_3,1_ = 35.79 Nm. For RFD, the following reliability statistics were collected: ICC_3,1_ = 0.389, SEM_3,1_ = 846.15 Nm∙s^−1^.

Torque signals were sampled at 1 kHz with a Biopac data acquisition system (MP150 Biopac Systems, Inc., Santa Barbara, CA, USA), recorded on a personal computer, and processed offline with custom written software (MATLAB, The MathWorks, Inc., Natick, MA, USA). PT was identified as the greatest torque achieved on the torque-time curve for each repetition. MT was defined as the average torque achieved on the torque-time curve for each repetition. RFD was defined as the greatest rate of change of force development over time between sampled data points. 

### 2.9. Statistical Analyses

Participants were separated into HI, MOD, and LO groups based on the muscle carnosine content of their VL. Participants with carnosine values lower than those corresponding to the 25th percentile were classified as “LO”, participants with carnosine values greater than those corresponding to the 75th percentile were classified as “HI”, and participants with carnosine values within the interquartile range were classified as “MOD”.

Differences in physical characteristics and anthropometrics, skeletal muscle carnosine content, and isometric and isokinetic performance between groups were performed with magnitude-based inferential analyses, which were used as an alternative to normal parametric statistics to account for the small sample sizes per group. Several studies have supported magnitude-based inferences as an alternative statistical tool to null hypothesis testing for reducing interpretation errors [22,23]. To make inferences about the true effects of muscle carnosine on the dependent variables, data were analyzed using magnitude-based inferences, calculated from 95% confidence intervals, as previously described [22]. Differences in dependent variables between groups as well as change scores in dependent variables were analyzed using the *p*-value from independent *t*-tests to determine a mechanistic inference utilizing a published spreadsheet [24]. Qualitative inferences were based upon the chances that the true magnitude of the effect of HI carnosine were substantially greater or smaller than MOD or LO values, and were assessed as: <1% almost certainly not; 1–5% very unlikely; 5–25% unlikely; 25–75% possibly; 75–95% likely; 95–99% very likely, and; >99% almost certainly [23]. If there was a greater than 5% chance that the true value was either greater or lesser, the effect was considered mechanistically unclear. The smallest non-trivial change, or smallest worthwhile change, was set at 20% of the grand standard deviation for all values [22]. Outliers were identified when values exceeded 1.5 times the interquartile range and were not included in the analysis [25]. All data are reported as mean ± standard deviation.

## 3. Results

### 3.1. Skeletal Muscle Carnosine

A total of 20 participants were included in the final data analysis, as one individual was removed from all analyses because she was considered to be an outlier, with respect to carnosine content. The mean skeletal muscle carnosine content for the 20 subjects was 6.35 ± 2.10 mmol·kg^−1^ wet weight (ww). The muscle carnosine content corresponding to the 25th percentile was 4.58 mmol·kg^−1^ ww, and that corresponding to the 75th percentile was 7.94 mmol·kg^−1^ ww. This resulted in a total of five subjects in the LO carnosine group, 10 subjects in the MOD carnosine group, and five subjects in the HI carnosine group. Magnitude-based inference analyses revealed that muscle carnosine content was most likely greater in HI compared to MOD, HI compared to LO, and MOD compared to LO (HI: 9.15 ± 0.98 mmol·kg^−1^ ww; MOD: 6.15 ± 1.19 mmol·kg^−1^ ww; LO: 4.02 ± 0.42 mmol·kg^−1^ ww).

### 3.2. Anthropometrics and Nutrient Intake

Group comparisons between age (HI: 22.2 ± 1.3 years; MOD: 23.3 ± 3.3 years; LO: 22.8 ± 1.9 years), body mass (HI: 62.8 ± 8.1 kg; MOD: 62.0 ± 9.2 kg; LO: 63.8 ± 3.9 kg), and body fat percentage (HI: 28.0 ± 5.0%; MOD: 26.6 ± 7.0%; LO: 26.0 ± 5.5%) were all unclear. However, group comparisons between height revealed that HI was likely taller than MOD, but differences between HI and LO and MOD and LO were both unclear (HI: 166.9 ± 5.2 cm; MOD: 161.2 ± 8.1 cm; LO: 166.9 ± 6.0 cm).

Dietary nutrient comparisons between groups for daily energy intake (HI: 1860 ± 634 kcal; MOD: 1498 ± 515 kcal, LO: 1357 ± 481 kcal) and daily fat intake (HI: 63.4 ± 23.9 g; MOD: 55.5 ± 20.1 g, LO: 47.7 ± 7.5 g) were unclear. However, group comparisons between daily carbohydrate intake revealed that HI had likely greater intake compared to LO, but differences between HI and MOD and MOD and LO were unclear (HI: 187.7 ± 43.9 g, MOD: 176.6 ± 74.7 g, LO: 124.9 ± 58.0 g). Additionally, HI had a likely greater daily protein intake compared to MOD and a very likely greater daily protein intake compared to LO, but the differences in daily protein intake between MOD and LO were unclear (HI: 102.2 ± 30.9 g; MOD: 73.5 ± 29.2 g, LO: 64.1 ± 8.5 g). When examining daily protein intake relative to body mass, the same results were found: HI had likely greater intake compared to MOD, very likely greater intake compared to LO, and the differences between MOD and LO were unclear (HI: 1.70 ± 0.45 g·kg^−1^, MOD: 1.17 ± 0.49 g·kg^−1^, LO: 1.00 ± 0.11 g·kg^−1^).

### 3.3. Isokinetic Performance

**Figure 2:**
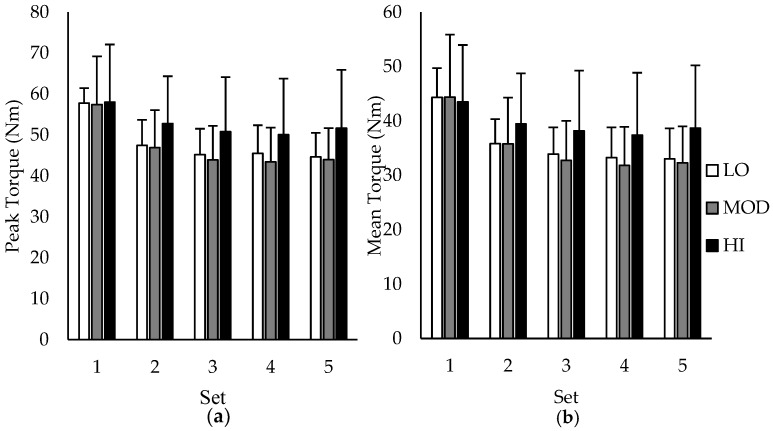
Average peak torque (**a**) and mean torque (**b**) from each set of the five sets of 50 isokinetic knee extension repetitions depending on intramuscular carnosine content. HI: high intramuscular carnosine content; MOD: moderate intramuscular carnosine content; LO: low intramuscular carnosine content. Standard deviations are denoted by positive error bars.

Group comparisons between average PT and MT over the five sets of 50 repetitions are depicted in Figure 2a,b, respectively. During sets 1–4, differences between groups in PT and MT were all unclear except for set 5, in which the MT for HI was likely greater than MOD. However, when examining group differences in change (∆) scores in PT and MT between sets, HI had a likely lower change in PT and MT compared to MOD from set 1–2, set 1–3, set 1–4, and set 1–5, indicating reduced fatigue in participants with the highest muscle carnosine content. Additionally, HI had a likely lower change in PT and MT compared to LO from set 1–3 and set 1–5. All other changes in PT and MT were unclear. Change scores in PT and MT based on intramuscular carnosine content are presented in Table 1 with mechanistic interpretations.

### 3.4. Isometric Performance

**Figure 3:**
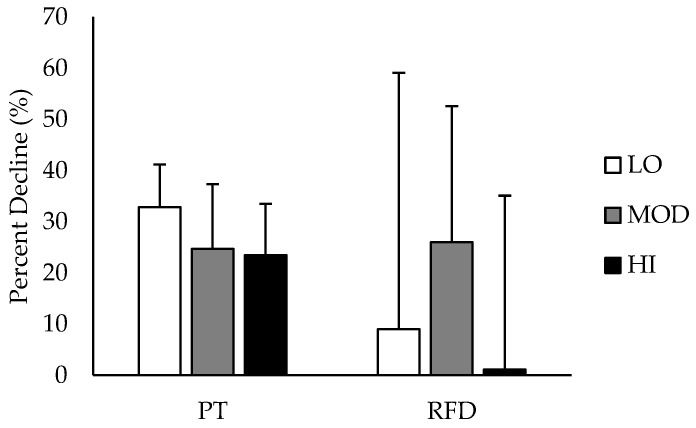
Average percent decline in peak torque and rate of force development during maximal voluntary isometric contraction of the knee extensors from before the muscle-fatiguing protocol to after, depending on intramuscular carnosine content. HI: high intramuscular carnosine content; MOD: moderate intramuscular carnosine content; LO: low intramuscular carnosine content; PT: peak torque; RFD: rate of force development. Standard deviations are denoted by positive error bars.

Comparisons between groups in % DEC in PT and RFD are depicted in Figure 3. % DEC in PT was likely greater in LO compared to HI, and % DEC in RFD was likely greater in MOD compared to HI. All other group differences in % DEC were unclear. Differences in % DEC in PT and RFD based on intramuscular carnosine content are presented in Table 2 with mechanistic interpretations.

## 4. Discussion

The results of this study indicate that recreationally trained women with HI intramuscular carnosine have a greater attenuation of fatigue during isokinetic and isometric exercise compared to recreationally trained women with lower intramuscular carnosine. Additionally, the findings of this investigation also indicate that women with the highest dietary protein intake were also more likely to have the highest intramuscular carnosine content.

Women who had the highest levels of intramuscular carnosine (seen in HI) were more likely to have a greater resistance to fatigue during the muscle-fatiguing protocol compared to MOD or LO. Additionally, subjects with HI carnosine had a greater attenuation of fatigue (as seen by the lower %DEC in PT and RFD) during the MVICs compared to those with LO or MOD, respectively. Because the changes in both MT and PT followed a similar pattern for each group between sets, these findings show that both PT and MT may be used interchangeably for quantification of muscle force output during isokinetic exercise. Our findings are consistent with other investigations showing that higher muscle carnosine is associated with lower fatigue rates. Previous research has demonstrated that β-alanine supplementation and the resultant increase in intramuscular carnosine is most beneficial for improving sporting performance during high-intensity exercise lasting between 1–4 min; this is because muscular contraction-induced acidosis is the primary contributing factor to fatigue during this time [26]. Therefore, having an increased carnosine content would help delay fatigue during activities that are limited by muscle acidosis. For example, Derave et al. [15] discovered that four weeks of 4.8 g·day^−1^ β-alanine supplementation resulted in increases in muscle carnosine content in trained sprinters and improved dynamic knee extension torque in the last two of five sets of 30 maximal isokinetic contractions, indicating that the increased muscle carnosine may have contributed to the decrease in fatigue. In addition, our findings are also consistent with Baguet and colleagues [8], who demonstrated that rowers with greater muscle carnosine content were able to sustain their rowing performance for a longer period of time compared to individuals with low carnosine. These results are supportive of evidence that carnosine may act as a Ca^2+^/H^+^ exchanger and H^+^ buffer, aiding in the removal of H^+^ from the active muscle and increasing the delivery of Ca^2+^ to the sarcomere, increasing force production and delaying fatigue [5,6,9]. These previous studies recruited primarily trained men to participate. This present investigation appears to be the first to demonstrate the fatigue resistance associated with elevated muscle carnosine levels in recreationally trained young women.

The muscle carnosine content of the participants investigated in the present study appear to be the highest among females reported in the literature accounting for dry muscle weight using the biopsy technique [10,11]. A possible explanation for this may be the high protein intakes of the subjects in the present study. When examining daily protein intake relative to body mass in our study, the average intake for the HI, MOD, and LO groups were all greater than the recommended daily allowance of 0.8 g·kg^−1^. Previous research has shown that intramuscular carnosine content is lower in vegetarians compared to omnivores [10,27,28]. Vegetarian diets are very low in β-alanine, which is primarily obtained from the diet through meat and fish [10]. In vegetarians lacking dietary meat, carnosine synthesis primarily occurs through hepatic production of β-alanine as a result of uracil degradation, limiting the amount of total carnosine produced [29]. Although none of the women in the previous investigations of Everaert and colleagues [10] and Mannion and colleagues [11] were reported to be vegetarians, it is likely that differences in daily protein intakes between those studies and the present investigation may have contributed to the differences reported in muscle carnosine. The present study recruited American women, while the other investigations involved European women. In general, dietary protein consumption is greater in Americans than it is in Europeans [30], which may provide reasoning behind the greater intramuscular carnosine content in our participants.

The results of this investigation also indicated that higher protein intakes (both absolute and relative intakes) were reflected by higher muscle carnosine levels. This is in contrast with Everaert et al. [10], who reported no difference in intramuscular carnosine content between omnivorous males who consumed high or low amounts of dietary β-alanine. However, our findings are in agreement with those of Jones [28], who reported that intramuscular carnosine content was greatest in Australian women consuming high amounts of meat products. These results indicate that greater dietary protein intake may result in greater carnosine content in women.

Another interesting finding of this investigation was that the differences in performance data between subjects with LO and MOD carnosine were all unclear. This may indicate that moderate and low levels of intramuscular carnosine affect performance to the same extent, but performance increases once a certain threshold of carnosine is met. Although many women in the LO group in our investigation had a greater carnosine content than those reported in the literature, the results of this study suggest that carnosine content may need to be elevated past a certain threshold to see performance improvements.

One potential limitation of the present investigation is that the research questions were examined using a cross-sectional design, limiting the findings. Because no experimental trial was used to examine the relationship between high protein intake, carnosine content, and exercise performance, our data is speculative, and further research should investigate the relationships between these variables. Additionally, there is a possibility that the differences in carbohydrate intake between groups (specifically, the greater carbohydrate intake in HI), and subsequently greater intramuscular glycogen content, may have played a role in the greater exercise performance in this group. Further research is warranted in this area.

## 5. Conclusions

In conclusion, the findings of this investigation show that high levels of skeletal carnosine may provide a degree of fatigue resistance during isokinetic and isometric performance in women. Additionally, our results also indicate that carnosine content in women appears to be influenced by dietary protein intake.

## Figures and Tables

**Table 1:** Group comparisons of average change scores in peak torque and mean torque from set 1 to all other sets of 50 isokinetic knee extension repetitions, with magnitude-based inferences and interpretations of mean differences.

						Percent Chance Greater			
Variable	Timepoint	Comparison	Group 1	Group 2	Mean Difference	Group 1	Trivial	Group 2	Interpretation
**Peak Torque (Nm)**	Set 1–2	LO vs. MOD	−10.3 ± 6.3	−10.5 ± 6.9	0.2 ± 8.0	30.9	42.0	27.1	Unclear
		LO vs. HI	−10.3 ± 6.3	−5.3 ± 6.6	−5.1 ± 9.4	5.9	18.5	75.6	Unclear
		MOD vs. HI	−10.5 ± 6.9	−5.3 ± 6.6	−5.3 ± 8.1	3.5	17.0	79.6	Likely Group 2
	Set 1–3	LO vs. MOD	−13.5 ± 7.5	−13.5 ± 8.1	−0.0 ± 10.0	32.9	33.7	33.4	Unclear
		LO vs. HI	−13.5 ± 7.5	−7.2 ± 5.9	−6.3 ± 10.0	4.9	13.7	81.4	Likely Group 2
		MOD vs. HI	−13.5 ± 8.1	−7.2 ± 5.9	−6.3 ± 8.9	3.1	13.2	83.7	Likely Group 2
	Set 1–4	LO vs. MOD	−12.2 ± 7.3	−14.0 ± 8.8	1.8 ± 9.9	47.1	32.2	20.6	Unclear
		LO vs. HI	−12.2 ± 7.3	−7.9 ± 6.5	−4.3 ± 10.0	9.1	22.4	68.5	Unclear
		MOD vs. HI	−14.0 ± 8.8	−7.9 ± 6.5	−6.1 ± 9.6	4.5	15.0	80.4	Likely Group 2
	Set 1–5	LO vs. MOD	−13.1 ± 6.8	−13.4 ± 8.9	0.3 ± 9.8	34.9	34.8	30.3	Unclear
		LO vs. HI	−13.1 ± 6.8	−6.4 ± 5.8	−6.7 ± 9.2	2.9	11.1	86.0	Likely Group 2
		MOD vs. HI	−13.4 ± 8.9	−6.4 ± 5.8	−7.0 ± 9.5	2.9	11.2	85.8	Likely Group 2
**Mean Torque (Nm)**	Set 1–2	LO vs. MOD	−8.5 ± 5.7	−8.6 ± 5.7	0.1 ± 6.8	28.6	44.8	26.6	Unclear
		LO vs. HI	−8.5 ± 5.7	−4.1 ± 5.5	−4.5 ± 8.2	5.5	19.2	75.4	Unclear
		MOD vs. HI	−8.6 ± 5.7	−4.1 ± 5.5	−4.6 ± 6.7	2.8	17.6	79.6	Likely Group 2
	Set 1–3	LO vs. MOD	−11.2 ± 7.1	−11.6 ± 7.0	0.4 ± 9.1	35.9	34.4	29.7	Unclear
		LO vs. HI	−11.2 ± 7.1	−5.3 ± 5.1	−5.9 ± 9.7	4.8	13.2	82.0	Likely Group 2
		MOD vs. HI	−11.6 ± 7.0	−5.3 ± 5.1	−6.3 ± 7.7	1.9	10.2	87.9	Likely Group 2
	Set 1–4	LO vs. MOD	−11.1 ± 7.1	−12.5 ± 7.4	1.5 ± 8.7	45.7	33.6	20.7	Unclear
		LO vs. HI	−11.1 ± 7.1	−6.1 ± 5.4	−4.9 ± 9.2	6.3	17.4	76.4	Unclear
		MOD vs. HI	−12.5 ± 7.4	−6.1 ± 5.4	−6.4 ± 8.1	2.3	10.4	87.3	Likely Group 2
	Set 1–5	LO vs. MOD	−11.3 ± 6.9	−12.1 ± 7.6	0.7 ± 8.8	38.8	34.9	26.3	Unclear
		LO vs. HI	−11.3 ± 6.9	−4.9 ± 4.6	−6.5 ± 8.6	2.7	10.1	87.2	Likely Group 2
		MOD vs. HI	−12.1 ± 7.6	−4.9 ± 4.6	−7.2 ± 8.1	1.5	7.6	90.9	Likely Group 2

“Group 1” refers to the first (left) group in the “Comparison column, whereas “Group 2” refers to the second (right) group in the “Comparison” column. “Mean Difference” represents the difference between “Group 1” and “Group 2”. Values for “Group 1”, “Group 2”, and “Mean Difference” are presented as mean ± standard deviation. “Percent Chance Greater” columns refer to the chance estimation that one group was greater than another for a particular variable, and the “Interpretation” column displays the mechanistic inference of which group had a greater value. HI: high intramuscular carnosine content; MOD: moderate intramuscular carnosine content; LO: low intramuscular carnosine content.

**Table 2:** Group comparisons of percent decline in peak torque and rate of force development during maximal voluntary isometric contraction of the knee extensors from before the muscle-fatiguing protocol to after, with magnitude-based inferences and interpretations of mean differences.

					Percent Chance Greater			
Variable	Comparison	Group 1	Group 2	Mean Difference	Group 1	Trivial	Group 2	Interpretation
**Peak Torque Percent Decline (%)**	LO vs. MOD	32.8 ± 8.4	24.7 ± 12.6	8.1 ± 14.0	81.7	12.2	6.2	Unclear
	LO vs. HI	32.8 ± 8.4	23.5 ± 10.0	9.3 ± 13.0	87.1	8.8	4.1	Likely Group 1
	MOD vs. HI	24.7 ± 12.6	23.5 ± 10.0	1.2 ± 14.0	43.9	26.0	30.2	Unclear
**Rate of Force Development Percent Decline (%)**	LO vs. MOD	8.96 ± 50.1	26.0 ± 26.6	−17.0 ± 61.0	18.0	16.6	65.3	Unclear
	LO vs. HI	8.96 ± 50.1	1.1 ± 34.0	7.9 ± 62.0	51.2	18.9	29.9	Unclear
	MOD vs. HI	26.0 ± 26.6	1.1 ± 34.0	25.0 ± 34.0	85.9	10.8	3.3	Likely Group 1

“Group 1” refers to the first (left) group in the “Comparison” column, whereas “Group 2” refers to the second (right) group in the “Comparison” column. “Mean Difference” represents the difference between “Group 1” and “Group 2”. Values for “Group 1”, “Group 2”, and “Mean Difference” are presented as mean ± standard deviation. “Percent Chance Greater” columns refer to the chance estimation that one group was greater than another for a particular variable, and the “Interpretation” column displays the mechanistic inference of which group had a greater value. HI: high intramuscular carnosine content; MOD: moderate intramuscular carnosine content; LO: low intramuscular carnosine content.
